# T254. IMPACT OF A DROP IN, OPEN ENDED, PSYCHO EDUCATIONAL GROUP ON CLIENT ENGAGEMENT IN AN EARLY PSYCHOSIS INTERVENTION PROGRAM

**DOI:** 10.1093/schbul/sby016.530

**Published:** 2018-04-01

**Authors:** Crystal Morris, Elham Sadeh, Greg McMillan, Sharman Robertson

**Affiliations:** The Ottawa Hospital

## Abstract

**Background:**

Participating in groups are a critical part of rehabilitation model in Early Psychosis Intervention. Effectiveness of intervention is dependent on the willingness of an individual to engage and remain engaged throughout the duration of the intervention. Approximately 30% of individuals with a first episode of psychosis disengage from services despite ongoing needs.

On Track is a community based mental health outpatient program specialized in early assessment & treatment of psychosis. Client population includes individuals aged 16 to 35 years residing in the North East of Ontario, Canada.

The program offers psychiatric follow up as well as unique group based interventions. Clients are required to be screened for group suitability & community safety prior to participation. Program evaluation highlighted challenges to engage clients. An open-ended, psycho-educational, drop in group called “The Café” was developed.

**Methods:**

Study used a mixed‐methods approach to evaluate the impact of the Café sessions on clients & their engagement in the program. Data was collected between December 2016 to June 2017. All clients were diagnosed with primary psychosis disorder. Patient experiences & engagement level were measured using a questionnaire & the Singh-O’Brien Level of Engagement Scale.

**Results:**

43 clients participated in 29 Café sessions in seven months. Average number of participants per session was 7.3 (SD=3.05). Over 72% of clients participated in at least one to five sessions. Average age was 23.9 (SD=0.6). Participants were predominantly male (82%), white (42%), English speaking (93%), living with their parents (76.2%), no employment (69%), with some college or university degree (45.2%). Primary source of income were their families (47.6%). Over 59% of participants were in the program one year or less, 18.2 % & 22.7 % of participants have been in the program for two & three years, respectively.

The engagement score among males did not differ significantly from females (t(42)=-0.94, p=0.35). There was no significant differences in engagement scores between ethnic groups (F(4)=2.84, p=0.062). There was a marginally significant difference in engagement score between program duration groups (F(2)=3.23, p=0.051); the engagement score for clients who have been enrolled in the program for over two years were slightly higher than one year clients.

41% of clients attended Café before participating in any other groups; 4 of them joined other recreational & leisure groups. 3 clients later enrolled in a skill based group. 92% of respondents commented about the benefit of Café to their recovery as being “good” or “better”. Qualitative data identified socializing, coping with illness, & drop in nature of the Café as main themes contributing to recovery.

**Discussion:**

In an attempt to increase opportunities for engagement, our program developed a drop in Café style group to provide client centered psycho-education group. The result of our intervention showed that the Café provided benefits to client’s engagement and recovery. The Café facilitated additional engagement in both recreational and leisure & skill based group streams. The drop in nature of the Café was advantageous to increasing engagement. Participation in the Café resulted in improvements in coping with the illness & socialization. Clients who had been enrolled in our program for 2 years reported higher levels of engagement compared to clients newly enrolled.

Evaluating our intervention through collecting both qualitative & quantitative data provided us with a better understanding of the impact of the open- ended, drop in nature, psycho-education based group. Involvement of both client’s and families in the identification of the topics was another advantage of our study.

